# Network meta-analysis explained

**DOI:** 10.1136/archdischild-2018-315224

**Published:** 2018-11-13

**Authors:** Sofia Dias, Deborah M Caldwell

**Affiliations:** 1 Centre for Reviews and Dissemination, University of York, York, UK; 2 Bristol Medical School, University of Bristol, Bristol, UK

**Keywords:** meta-analysis, evidence based medicine, statistics

## What is network meta-analysis?

Healthcare decisions should be based on all relevant evidence.^1^ Usually, this is provided by randomised controlled trials (RCTs) comparing two or more interventions for a condition affecting a target population of interest, although other forms of evidence can be considered.^1 2^ When more than one study is available, meta-analysis can be used to combine multiple treatment effects and obtain an overall estimate of the effect in the target population. To assess clinical effectiveness, evidence from RCTs is typically used and relative treatment effects estimated in individual trials are pooled using methods that preserve within-trial randomisation. However, for the majority of health conditions, there are more than two interventions of interest. In such cases, performing multiple pairwise meta-analyses (comparing interventions two at a time) or lumping every active intervention to be compared with a ‘control’ is of limited use for decision-making and does not allow for coherent and transparent decisions. Decisions involving >20 interventions are not uncommon.^3–6^ The number of pairwise comparisons required to make a decision between 3 interventions is 3, with 5 interventions it is 10, with 10 interventions it is 45 and with 41 interventions^4^ it is 820. Clearly, not all comparisons will have been carried out in RCTs but looking at multiple separate pairwise analyses carried out using different sets of trials makes it impossible to decide which intervention is best.

Network meta-analysis (NMA), also termed multiple treatment meta-analysis or mixed treatment comparisons, was developed as an extension of pairwise meta-analysis to allow comparisons of more than two interventions in a single, coherent analysis of all the relevant RCTs.^7–10^ Its main advantages are that it produces consistent estimates of the relative effects of all interventions compared with every other in a single analysis using both direct and indirect evidence, while also correctly incorporating the relative effects from trials with more than two arms (ie, avoiding double counting of patients). This results in greater precision of treatment effect estimates and the ability to rank all the interventions in a coherent way.^8^ As for standard meta-analysis, NMA can be performed for most types of RCT outcomes, continuous, dichotomous, event rates and from survival models, using an appropriate scale (mean difference (MD), OR, relative risk, HR, etc).

The underlying idea is very simple: consider three friends, Anne, Ben and Charles. If we know that Ben is 7 cm taller than Anne, and that Charles is 10 cm taller than Anne, then we know that Charles is 3 cm taller than Ben, and is therefore the tallest. We can also rank the friends in terms of who is tallest as 1=Charles, 2=Ben, 3=Anne. So, by taking Anne’s height as reference and measuring the heights of the others compared with hers, we know how everyone’s height compares to each other and how to order the friends by height. The only assumption being made is that the heights we measured are an accurate reflection of the true heights of the three friends (in other words, we used a sufficiently accurate measuring tool). It is easy to see that the same relative heights and ranks would be obtained if one of the male friends had been the reference, and how the height relationships would extend if more than three friends had been measured. This is exactly how NMA works, although we also take the uncertainty (ie, the sampling error) in the relative effect estimates into account, as is standard in meta-analysis.

**Figure 1:**
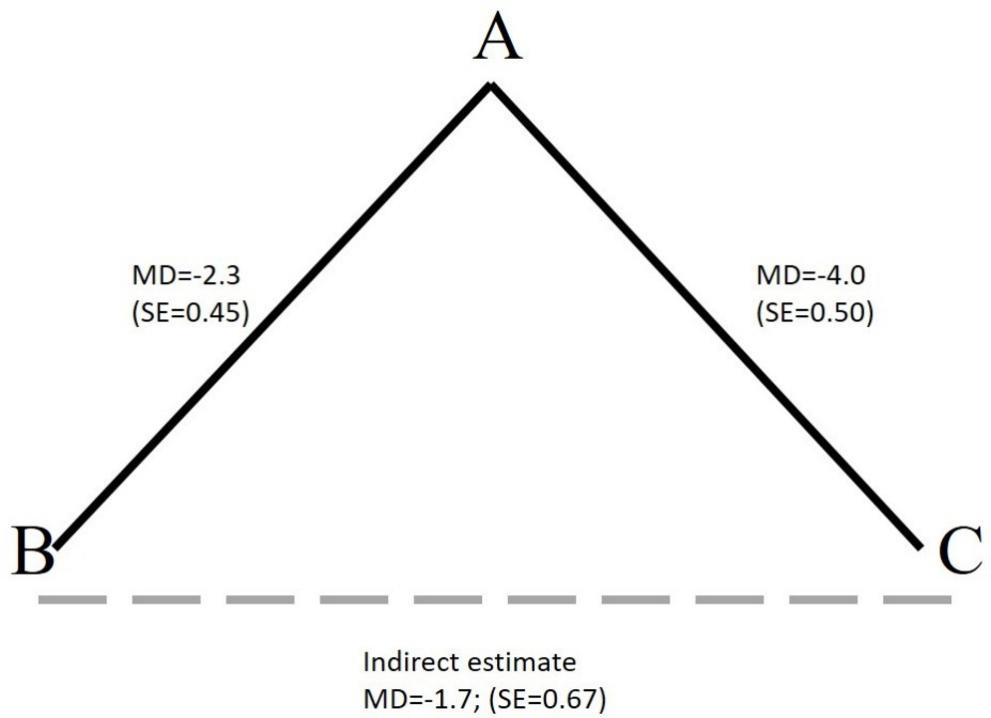
An example of a network of three treatments compared in two trials (solid black lines), where an indirect comparison can be made (dashed grey line). MD, mean difference.

Suppose we are interested in comparing treatments B and C. We find one trial comparing B with A, giving an MD of −2.3 with an SE of 0.45 and one trial comparing C with A giving an MD of −4 with an SE of 0.5. This suggests that both treatments B and C are better than A with 95% CIs that exclude no effect: (−3.18 to –1.42) for B compared with A and (−4.98 to –3.02) for C compared with A (assuming a reduction in the mean is desirable, eg, for pain). The network formed by these comparisons is given in figure 1. In the absence of a direct RCT, what can we say about the relative effect of treatment C versus B? The first thing to note is that this question can only be answered in the context of a predefined patient population of interest. That is, one must ensure that the populations included in the B versus A and the C versus A trials are comparable to each other and to our target population, with respect to any potential effect modifying characteristics.^10^ Once it is decided that these studies were carried out on clinically homogeneous populations (which, in turn, are similar to the target population) then it can be assumed that each study estimates the true treatment effects in the target population (ie, the relative effects were measured using an ‘accurate measurement tool’). We can therefore say that C is better than B by 1.7 units through the *indirect comparison* of treatments C and B,^11^ since the true treatment effects of B versus A, C versus A and C versus B must be *consistent*, that is, they must add up in the same way as the friends’ heights. In other words, the MD of C compared with B must be the difference between the MDs of C versus A and B versus A, which is −1.7 units. This consistency relationship, sometimes also called transitivity, must hold if the studies are estimating the *true* effects in a patient population. To obtain a 95% CI for the C versus B comparison, we simply add the variances of the two other comparisons to obtain an SE with which a 95% CI can be constructed: (−3.01 to –0.39). This type of comparison is termed ‘indirect’ as it relies on evidence against the comparator A, and not on ‘direct’ head-to-head evidence from one or more trials of C versus B.

Suppose now that we also had evidence from a new study on the same patient population which compared treatment C with B, giving an MD of −1.8 95% CI (−3.66 to 0.06). Traditional hierarchies of evidence state that estimates from direct head-to-head RCTs provide the ‘best available’ evidence of intervention effects. Should we now discard the indirect evidence? Or perhaps we should prefer the indirect evidence since it suggests a statistically significant effect? To do either is contrary to the principle of using all relevant evidence for decision making.^1 12^ The solution is to use both the direct and indirect evidence to do a mixed (evidence) treatment comparison, or NMA, under the assumption that both the direct and indirect evidence are estimating the same, true, underlying treatment effect of C versus B in our target population. The exact same principles apply when multiple RCTs are available for each of the possible comparisons and for more than three interventions.

NMA relies on the same assumptions underlying pairwise meta-analysis, that is, the included studies are sufficiently homogenous in terms of the condition being studied, the included participants and the definition of active and control interventions. In other words, we are assuming that the effects of B versus A and C versus B that would have been observed if the C versus A RCT had included all three treatments, is the same as that observed in the B versus A RCT (apart from sample variability). This assumption is the basis for coherent decisions whether they involve two or more treatments. One way to empirically check this is to ask: ’given the known study and participant characteristics, if all these studies compared the same two treatments, would it be suitable to combine them in a meta-analysis?' If the answer to this is yes, and the only distinction is that instead the studies compare different sets of interventions, then the assumption of ‘sufficient homogeneity’ is, in principle, satisfied.

**Figure 2:**
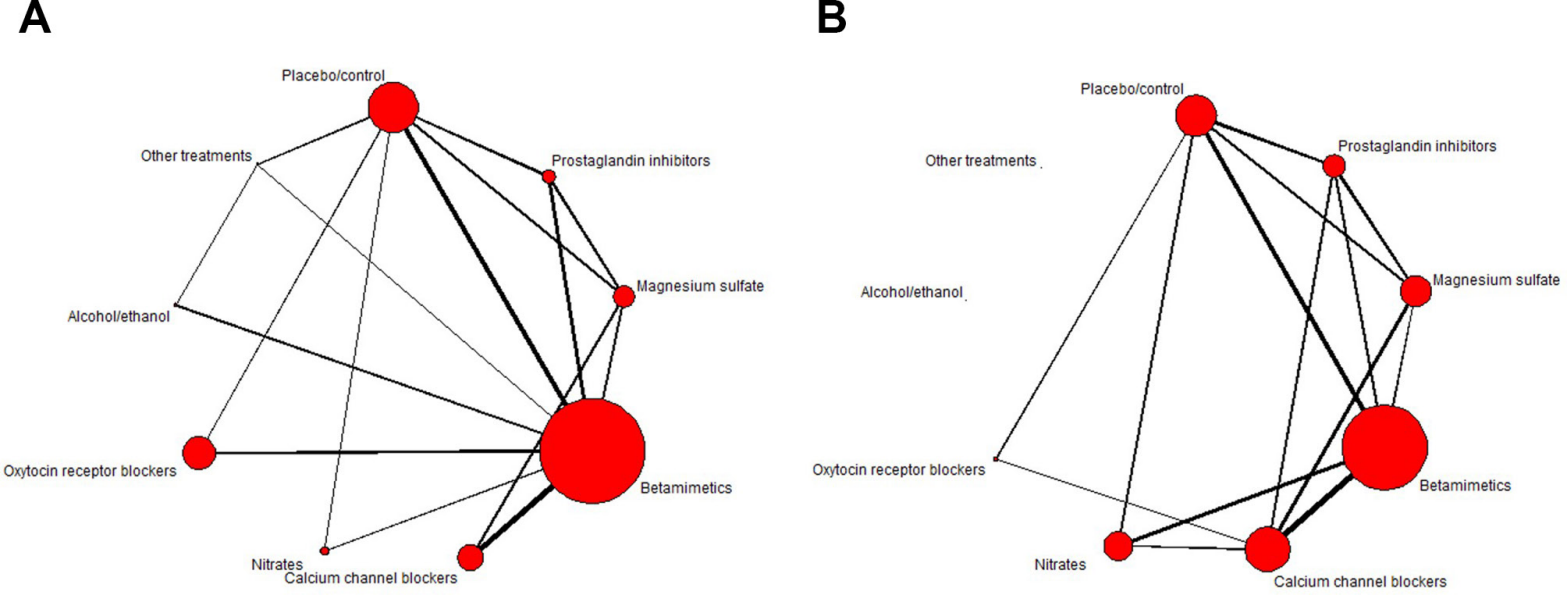
Network plots for (A) perinatal death and (B) estimated gestational age at delivery. The size of the circles is proportional to the number of patients randomised to each intervention and width of the lines is proportional to the number of studies making each comparison. Data from the National Institute for Health and Care Excellence guideline.^13^

Because NMA pools the relative treatment effects estimated across RCTs, within-trial randomisation is preserved. As long as the interventions of interest form a connected network of comparisons, then relative effects of each intervention compared with every other can be obtained, along with estimates of their uncertainty (eg, 95% CIs). Figure 2 shows two networks of tocolytic interventions compared in a guideline produced for the National Institute for Health and Care Excellence,^13^ which updated a previously published systematic review and NMA.^14^ Nine types of tocolytic interventions were of interest (table 1) and 98 RCTs were included. To ensure that the assumption of consistency of treatment effects was reasonable, the study characteristics and possible effect modifiers were first assessed by the reviewers.^14^ For example, the analysis excluded trials in which women were at high risk of preterm delivery such as those with multiple gestation and ruptured membranes. Underpinning this exercise is the need to determine if every individual included in every trial across the network could have been (hypothetically) randomised to any of the included treatments. If, for example, a treatment would only be administered to a multiparous and not nulliparous woman or as a second-line or third-line treatment, it is possible that the assumption of consistency might not hold.

**Table 1:** Tocolytic therapies of interest

	Interventions
1	Placebo/control
2	Prostaglandin inhibitors
3	Magnesium sulfate
4	Betamimetics
5	Calcium channel blockers
6	Nitrates
7	Oxytocin receptor blockers
8	Alcohol/ethanol
9	Other treatments

The first network (figure 2A) includes 47 RCTs reporting on perinatal death. It is ‘connected’ as there is a path that connects every treatment and therefore all relative effects for all interventions compared with every other can be obtained. We can see that the interventions with the most patients randomised are (in decreasing order) betamimetics and placebo, as they have the widest circles. The second network, (figure 2B), includes the 51 RCTs which reported on estimated gestational age (EGA) at delivery. This figure has two disconnected interventions: ‘alcohol/ethanol’ and ‘other treatments’ cannot be compared with the rest of the network. For this outcome, comparisons can only be made between the other interventions. More information can be added to these plots to show other network characteristics,^14 15^ although the ability to display additional information can be limited by the size of the network.

## NMA models and estimating treatment effects

NMA simultaneously combines the relative treatment effects estimated within each study while accounting for the individual treatments being compared and correctly incorporating studies with more than two arms. Fixed or random effects models can be fitted, the latter allows for between-study heterogeneity. NMA random effects models usually assume that the between-study heterogeneity is the same across all comparisons, that is, a single measure of heterogeneity is calculated across the whole network, although models allowing for different heterogeneity for each comparison can also be fitted.^16^ In the presence of large heterogeneity, patient or study characteristics that may modify the relative treatment effects (effect modifying covariates) should be investigated using meta-regression^17^ or sensitivity analyses.

As a statistical model, NMA can be fitted using a frequentist or Bayesian approach.^18^ A Bayesian approach to NMA requires a prior probability distribution to be specified for the parameter of interest (eg, treatment effect), describing the range and probability of plausible values for the parameters, which is combined with a likelihood statement, which describes the data collected, using Bayes theorem. This produces a posterior distribution which describes the new range and probability of plausible values for the parameter, on which statistical inferences are based.^19^ Uncertainty in the parameters is fully represented by their posterior distributions, so direct probability statements can be made on, for example, the probability that treatment X increases EGA at delivery by 4 weeks. Consequently, Bayesian NMAs are more commonly used as they naturally produce ranking and probability outputs useful for decision-making and allow for greater flexibility in the models fitted.^10^ For example, a ‘class model’ can be used where interventions that have a similar mode of action (ie, belong to the same ‘class’) are assumed to have similar, but not identical, effects compared with the reference treatment as used in the tocolytics example^13 14^ (figure 2 shows the networks at class level). This allows an overall decision to be made at a class level, but individual treatment profiles and costs can also be incorporated in clinical or health economic decisions.

When data are sparse, for example, for adverse or rare events, Bayesian methods have additional advantages such as the ability to better handle studies with zero cells and the potential for including any relevant prior information. However, for most NMAs only simple models are required and no prior information is used, with Bayesian approaches typically defining non-informative prior distributions for all treatment effect parameters, making results from frequentist or Bayesian analyses very similar. The main difference between these approaches is how results are presented. Results from a frequentist NMA will be presented as estimated relative effects (eg, MD, OR, etc) and a 95% CI, whereas results from a Bayesian analysis will be presented as summaries from the posterior distribution of the MD (or OR), which can be the mean or median MD (OR, etc) and their 95% credible interval (CrI).^15^ A Crl is interpreted as the interval where there is a 95% probability that the values of the MD (OR, etc) will lie. Medians are recommended for ratio measures such as the OR, HR or risk ratio, whereas either the mean or median can be reported for the MD or standardised MD.

Regardless of the framework used, the fit of the model to the data should be assessed, and in networks with both direct and indirect evidence contributing to estimates, the assumption of consistency should also be statistically assessed. This can be done by comparing the results obtained using the direct evidence alone with the results obtained using the indirect evidence alone,^20 21^ and calculating a p value for their difference. Methods that assess consistency in the whole network^22 23^ are also available and often preferred when networks are large.^10^ Should evidence of inconsistency be found, the characteristics of all included studies should be re-examined and adjustment for effect modifiers (eg, covariates or risk of bias) should be considered.

## Reporting and interpreting results

Table 2 reports the MDs in EGA (in weeks) with their 95% CrIs. Colloquially known as a ‘triangle table’, it displays findings from different analyses, in this case from the direct, pairwise analyses and the NMA analysis. Values shown in the upper diagonal are the MDs for the column header versus the row header and are derived from the NMA, and values in the lower diagonal are the MDs for the row header versus the column header. This ensures that the values are easily comparable across the two analyses. For example, the MD estimated from the NMA (upper diagonal) for prostaglandin inhibitors versus placebo is 2.32 (95% CrI 1.27 to 3.35), suggesting that there is evidence that the intervention increases EGA at delivery, by 2.3 weeks, compared with placebo. In addition, we can say that there is a 95% probability that this increase is between 1.27 and 3.35 weeks. We can compare this with the MD estimated from the direct evidence alone (lower diagonal) 3.27 (95% CrI 1.68 to 4.78). The first thing to note is that the point estimates are close and the 95% CrIs overlap considerably. However, the 95% CrIs from the NMA are more precise. In contrast, the MD of magnesium sulfate compared with placebo estimated from the direct evidence and the NMA appear contradictory, with estimates in opposite direction, although the very wide 95% CrI for the pairwise meta-analysis overlaps with those obtained from the NMA, which are also narrower. The consistency assumption was checked^13^ and some evidence of conflict between direct and indirect evidence was identified for this comparison (indirect OR=1.29 (95% CrI 0.29 to 2.27), p=0.015). No explanation was found for this conflict, but it was taken into account when making decisions.

**Table 2:** Mean differences and 95% CrI for EGA at delivery (in weeks) from the pairwise and network meta-analyses

·	Placebo/control	Prostaglandin inhibitors	Magnesium sulfate	Betamimetics	Calcium channel blockers	Nitrates	Oxytocin receptor blockers
Placebo/control		2.32 (1.27 to 3.35)	1.29 (0.29 to 2.27)	1.25 (0.40 to 2.07)	1.69 (0.69 to 2.66)	1.65 (0.52 to 2.78)	0.68 (−1.32 to 2.67)
Prostaglandin inhibitors	3.27 (1.68 to 4.78)		−1.04 (−2.01 to –0.04)	−1.08 (−2.08 to –0.05)	−0.64 (−1.68 to 0.42)	−0.67 (−1.97 to 0.67)	−1.65 (−3.76 to 0.52)
Magnesium sulfate	−0.14 (−1.60 to 1.28)	−0.23 (−1.45 to 0.97)		−0.04 (−0.99 to 0.91)	0.40 (−0.51 to 1.31)	0.36 (−0.88 to 1.63)	−0.61 (−2.69 to 1.50)
Betamimetics	1.91 (0.90 to 2.90)	−1.56 (−3.42 to 0.28)	−0.19 (−2.78 to 2.45)		0.44 (−0.32 to 1.20)	0.40 (−0.54 to 1.37)	−0.57 (−2.58 to 1.47)
Calcium channel blockers	na	−0.53 (−2.32 to 1.25)	−0.02 (−1.25 to 1.22)	0.80 (−0.08 to 1.67)		−0.03 (−1.16 to 1.10)	−1.01 (−2.98 to 0.99)
Nitrates	0.17 (−1.72 to 2.06)	na	na	−0.58 (−0.47 to 1.67)	1.30 (−1.07 to 3.68)		−0.98 (−3.15 to 1.21)
Oxytocin receptor blockers	0.90 (−1.74 to 3.53)	na	na	na	−1.21 (−3.66 to 1.23)	na	

The upper diagonal displays the mean differences for the column intervention vs the row intervention, derived from the NMA. Values >0 favour the column defining intervention. The lower diagonal displays the mean differences for the row intervention vs the column intervention, derived from direct comparisons only. Values >0 favour the row defining the intervention.

Adapted from the National Institute for Health and Care Excellence guideline.^13^

Crl, credible interval; EGA, estimated gestational age; na, not available; NMA, network meta-analysis.

The empty cells in the lower diagonal denote that no direct evidence was available for that comparison (eg, calcium channel blockers vs placebo), whereas the NMA can make all the comparisons and show that there is evidence of an increase in EGA at delivery for all interventions compared with placebo, except oxytocin receptor blockers (MD 0.68, 95% CrI −1.32 to 2.67).

Triangle tables can also be used to report two different outcomes, with one in the top half and the other in the bottom half. This can be a good way to display results from two important outcomes, for example, effectiveness and acceptability^24^ or other closely related outcomes,^4^ although it can be limited by the required width when there are many interventions. Similar vertical displays can convey the same information^14 15^ and relative effects can also be presented as forest plots.^13 14^

Table 3 shows treatment rankings and the probability that each intervention (or class) is the ‘best’ or in the top three for EGA at delivery. Here ranks are reported for effectiveness, such that rank 1 means the intervention is most effective. Placebo has a mean rank of 6.74 and a median rank of 7 (95% CrI 6 to 7). That is, on average, placebo was ranked approximately seventh out of seven treatments (ie, worst) for gestational age at delivery. Conversely, prostaglandin inhibitors were ranked first out of all seven treatment classes and had a 74% probability of being the most effective treatment and a 97% probability of being in the top three treatment classes to increase EGA.

**Table 3:** Posterior rank statistics and probabilities for the outcome EGA at delivery

Interventions	Rank statistics			Probabilities	
	Mean	Median	95% CrI	Best	Top 3
Placebo/control	6.74	7	(6 to 7)	0.00	0.00
Prostaglandin inhibitors	1.38	1	(1 to 4)	0.74	0.97
Magnesium sulfate	4.26	4	(2 to 6)	0.01	0.28
Betamimetics	4.48	5	(2 to 6)	0.00	0.16
Calcium channel blockers	2.84	3	(1 to 5)	0.07	0.76
Nitrates	3.04	3	(1 to 6)	0.13	0.65
Oxytocin receptor blockers	5.27	6	(1 to 7)	0.05	0.19

An alternative way of reporting ranks is to consider the cumulative probabilities using the surface under the cumulative ranking curve (SUCRA),^25^ which transforms the cumulative probabilities into a single value between 0 and 1, where a larger value indicates the more effective treatment. This is often reported as a percentage.^15^

All probabilities, SUCRA values and rankings should be interpreted with caution as they are very sensitive to the uncertainty in the relative treatment effects used to produce them. Measures or displays which capture this uncertainty such as a table of rank statistics with 95% CrI (table 3), rank probability plots (rankograms)^10 14 25^ or cumulative ranking probability plots^15 25^ should be reported in preference to single values such as the probability of being best or SUCRA. It is also imperative that ranking results are considered alongside the estimates of relative treatment effects as a treatment could have a higher rank without evidence of having a better effect than any of the others.

Importantly, all results should be interpreted taking into account the uncertainty in the estimates (conveyed by the 95% CrI) as well as the risk of bias in the included evidence. Tools that allow an examination of the impact of studies at risk of bias,^26 27^ and the impact of changes in the evidence on the decision^28^ have been developed and can help to interpret the findings from an NMA.

## Conclusions

When more than two interventions are being considered, synthesis of RCTs using an NMA will ensure that all the relevant evidence, whether direct or indirect, is used to produce coherent estimates of the relative effects of every intervention compared with every other. This allows for more efficient use of the relevant evidence, which can increase the precision of the estimates. In addition, because multiple sources of evidence are used, the final estimates are more robust than if only direct sources of evidence were included, that is, they are less likely to be influenced by the inclusion or exclusion of a single trial. The underlying assumption is that there are no participant or study characteristics that would modify the relative treatment effect of each treatment compared with every other.

Relying on multiple pairwise meta-analyses, each including a different set of trials may lead to incoherent decisions and does not make the best use of the available evidence.

It is important to display NMA results carefully to aid interpretation and to clinically and statistically assess the plausibility of the assumptions made.
